# Population divergence time estimation using individual lineage label switching

**DOI:** 10.1093/g3journal/jkac040

**Published:** 2022-02-15

**Authors:** Peter Beerli, Haleh Ashki, Somayeh Mashayekhi, Michal Palczewski

**Affiliations:** 1 Department of Scientific Computing, Florida State University, Tallahassee, FL 32306, USA; 2 Foundation Medicine Inc, San Diego, CA 92121, USA; 3 Department of Mathematics, Kennesaw State University, Marietta, GA 30060, USA; 4 Maplebear Inc., San Francisco, CA 94105, USA

**Keywords:** coalescence, gene tree, species tree, Bayesian inference, divergence time

## Abstract

Divergence time estimation from multilocus genetic data has become common in population genetics and phylogenetics. We present a new Bayesian inference method that treats the divergence time as a random variable. The divergence time is calculated from an assembly of splitting events on individual lineages in a genealogy. The time for such a splitting event is drawn from a hazard function of the truncated normal distribution. This allows easy integration into the standard coalescence framework used in programs such as *Migrate*. We explore the accuracy of the new inference method with simulated population splittings over a wide range of divergence time values and with a reanalysis of a dataset of 5 populations consisting of 3 present-day populations (Africans, Europeans, Asian) and 2 archaic samples (Altai and Ust’Isthim). Evaluations of simple divergence models without subsequent geneflow show high accuracy, whereas the accuracy of the results of isolation with migration models depends on the magnitude of the immigration rate. High immigration rates lead to a time of the most recent common ancestor of the sample that, looking backward in time, predates the divergence time. Even with many independent loci, accurate estimation of the divergence time with high immigration rates becomes problematic. Our comparison to other software tools reveals that our lineage-switching method, implemented in *Migrate*, is comparable to *IMa2p*. The software *Migrate* can run large numbers of sequence loci (>1,000) on computer clusters in parallel.

## Introduction

In phylogenetics and population genetics, often we need to know the time when populations split and evolved independently or when populations started to have reduced gene flow among them; Wakeley and Hey (1997) define an *isolation* model (I) in which the divergence marks the time when the ancestral population split into 2 groups of individuals that stop exchanging genetic material with each other. Given that most populations within a species may still exchange migrants after divergence, this definition seems overly strict. Consequently, Nielsen and Wakeley (2001) developed the *isolation with migration* (IM) model; in their model, the divergence time marks the change from a panmictic ancestral population to 2 populations linked by gene flow. In both models, the divergence times of the populations is always predated by the divergence time of the genes (cf Edwards and Beerli 2000; Arbogast et al. 2002). Both the isolation model and the isolation-with-migration model became popular and were implemented in several software packages: for example *IMa* (Hey 2010), *Lamarc* (Kuhner 2006), and BEAST 2 (Bouckaert et al. 2014) implemented the IM model, whereas *BPP* (Yang and Rannala 2010) and *Momi2* (Kamm et al. 2020) implemented the isolation model with admixture events.

The isolation with migration model treats the divergence time as a boundary between 2 models: a structured coalescent population with migration and a panmictic, ancestral population. We describe here an approach that combines migration and divergence within the same structured coalescence framework allowing the boundary to be more fluid. The extent of the boundary is defined by 2 parameters, the mean of the distribution of the boundary, the divergence time, and the standard deviation of the boundary. We implemented the new method in the program *Migrate* (Beerli 2006), which was used for all evaluations in this research. The MIT-licensed, open source software *Migrate* is available from the website http://popgen.sc.fsu.edu.

## Materials and methods

All current coalescence-based methods for estimating a divergence time *τ* between 2 populations treat the time as a boundary between 2 different models: the panmictic, ancestral population modeled using the single population coalescent and a population with 2 subpopulations using the structured coalescent with migration. In a Bayesian inference method, the boundary is adjusted using a prior distribution.

**Fig. 1.**
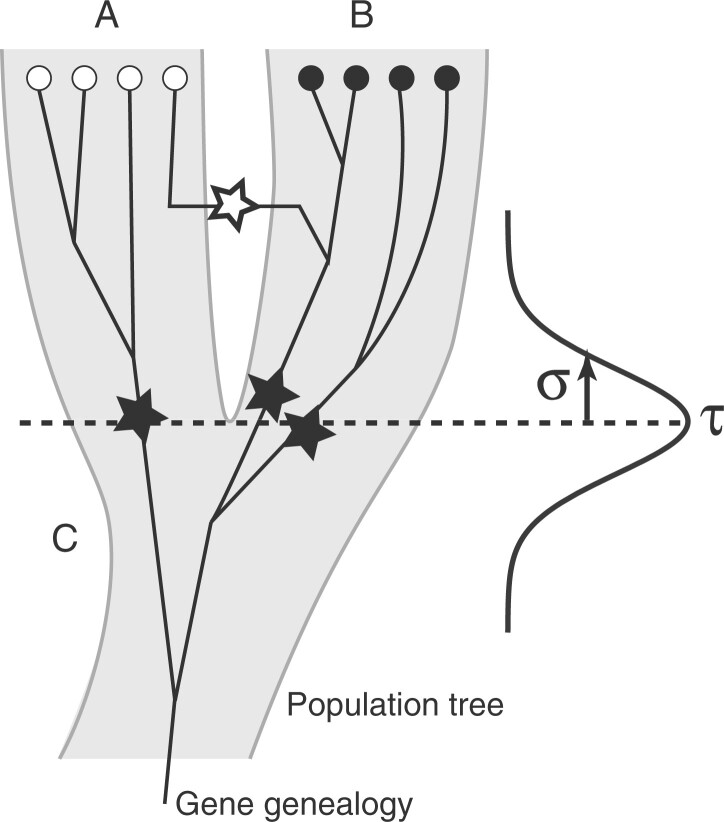
Visualization of population splitting of 2 populations A and B split at times *τ*, lineages in A and B split from the ancestral population C: the divergence time is based on individual lineage population label switching events (dark star) drawn from a distribution with mean (*τ*) and standard deviation (*σ*); migration events (white star) are drawn from the standard structured coalescent.

Here, we propose a different model. We consider the divergence time as a random variable with a normal distribution. The mean and standard deviation of this distribution are unknown and estimated. We use this distribution to draw times for divergence events for each lineage. We assume that we know the population or species label of the sampled individuals. Looking backward in time, each sample lineage will be at risk to switch labels irreversibly from a “derived” to an “ancestral” state. Therefore, at a given time in the genealogy, some lineages are in the ancestral population and some are not. Figure 1 shows an example with a divergence times *τ*. If we assume that this time is fixed, then the figure represents the isolation-with-migration model. If we assume that we have a normal distributed divergence time with parameters *τ* and *σ*, then individual lineages can change their state from the “descendant” state to the “ancestor” state by drawing times from this distribution and inserting a “divergence” event. This process is similar to how migration events are drawn.

### Hazard functions and waiting times

Population genetic inferences commonly use a sample of individuals collected recently, and we are interested in the potential interactions of these individuals in the past. The coalescent (Kingman 1982) and its extensions, such as those described by Strobeck (1987) and Hudson (1991), allowed probabilistic reconstruction of potential genealogies of the sample taking into account population sizes, migration rates, and other population genetic quantities. For example, in population parameter inference by Beerli and Felsenstein (2001) and Beerli (2006), the times of coalescence events and migration events are drawn from an exponential distribution with a rate that is defined by parameters for population size, migration rate, and the number of sampled lineages in each population. Looking backward in time, a sample lineage is at risk of a coalescence event or a migration event. The risk of coalescence or immigration, switching population labels, in this framework is constant for a given sample size. We can think of this process as a hazard that the lineages will fail to maintain their current status. The *hazard function* is commonly used in an engineering or survival context, where the condition of an object can suddenly change at any given time; the hazard can be constant, increasing, or decreasing with time. The function is expressed as a ratio of the probability density function f(·) and the complement of its cumulative density function F(·). For the exponential probability density function, this hazard is a constant, and for the Kingman coalescent, this is

(1) $$\begin{array}{l} \lambda_{c_{i}}=\frac{f(t)}{1-F(t)}=\frac{\lambda_{c_{i}}e^{-t\lambda_{c_{i}}}}{1-\int_{0}^{t}\lambda_{c_{i}}e^{-t\lambda_{c_{i}}dt}}\\ \;\;\;\;\;\;\;\;\;\;\;\;\;\;\;\;\;\;\;\;\;\;\;\;\;\;\;\;\;\;\;\;\;\;=\frac{\frac{k_{i}(k_{i}-1)}{\Theta_{i}}e^{-t\frac{k_{i}(k_{i}-1)}{\Theta_{i}})}}{1-\int_{0}^{t}\frac{k_{i}(k_{i}-1)}{\Theta_{i}}e^{-t\frac{k_{i}(k_{i}-1)}{\Theta_{i}}}dt}\\ \;\;\;\;\;\;\;\;\;\;\;\;\;\;\;\;\;\;\;\;\;\;\;\;\;\;\;\;\;\;\;\;\;=\frac{k_{i}(k_{i}-1)}{\Theta_{i}}. \end{array}$$

where Θ_*i*_ is the mutation-scaled effective population size and *k_i_* is the number of lineages in population *i* at time *t* (Wright 1951; Kingman 1982).

In the structured coalescence, migration is treated similarly. An immigration event happens at the rate *M_ji_* for each lineage, where *M_ji_* is the mutation-scaled immigration rate from population *j* into *i*, which is equivalent to the immigration rate *m_ji_* divided by the mutation rate *μ*. The total rate of migration is the sum of all possible migration rates *M_ji_* over all the lineages that have not yet coalesced:

(2) $$\lambda_{M_{.i}}=\sum_{j=1}^{n_{p}}k_{i}M_{ji},$$

where *n_p_* is the number of populations and *k_i_* is the number of lineages in population *i* at time *t*. Since the rate of immigrations and coalescences $\lambda_{c+m}=\sum_{i}^{n}\lambda_{c_{i}}+\lambda_{M_{.i}}$ is independent of the waiting time *t* which elapses before a coalescent or migration event happens in the interval $\left[t_{0},t_{0}+u\right]$. It has a probability density function of the exponential mixture

(3) $$f_{c+m}(u|\Theta ,M)=e^{-\int_{t_{0}}^{t_{0}+u}\lambda_{c+m}dt}\lambda_{c+m}=e^{-u\lambda_{c+m}}\lambda_{c+m}.$$

### Divergence time as events on lineages

In 2000, Nielsen and Slatkin introduced and later (Hey and Nielsen 2007) refined a model that adds population splitting, thus removing the assumption that populations are present for a very long time without removing the assumption of migration between the populations. We have developed an alternative to Nielsen’s and Hey’s approach that allows distributing the analysis onto cluster computers and using large datasets.

We treat the time of splitting as a random variable with a particular probability density. We chose to use the zero-truncated normal distribution because it has 2 parameters: mean and standard deviation. These parameters are commonly used and discuss quantities of interest. The mean describes the most likely time of the population divergence and the standard deviation describes the uncertainty of that divergence time. We consider the normal distribution a good choice to discuss divergence times, but it certainly is not the only possible distribution. We could have used a Weibull distribution or a Gamma distribution, both have a natural bound at zero, but their standard parameters are less familiar to biologists.

Looking backward in time, we know the fate of a lineage sampled today; any individual sampled today must have started out in an ancestral population; thus, each lineages sampled today is at risk to switch from the derived to the ancestral population. We assume that the risk of failure to stay in the derived population is increasing the further back in time the process moves. This process can be expressed with a hazard function of the normal distribution that is not constant, in contrast to the hazard function of the exponential distribution. The use of this hazard function allows us to integrate our population splitting distribution into our coalescence with migration framework. To express the risk of switching the population label (population splitting) we calculate the rate of splitting (divergence) events $\lambda \prime_{d}(t)$ by using the hazard function of a truncated normal distribution with bounds $b_{0}=0.0$ and *b*_1_ as

(4) $$\lambda \prime_{d}(t)=\lambda_{N(\mu ,\sigma )}(t)=\frac{\sqrt{\frac{2}{\pi }}e^{-\frac{{\left(\mu -t\right)}^{2}}{2\sigma^{2}}}}{\sigma (\text{erf}\left(\frac{\mu -t}{\sqrt{2}\sigma }\right)-\text{erf}\left(\frac{\mu -b_{1}}{\sqrt{2}\sigma }\right))\prime }$$

*μ* and *σ* are the parameters of the normal distribution; erf is the error function

(5) $$\text{erf}(x)=\frac{2}{\sqrt{\pi }}\int_{0}^{x}e^{-t^{2}}dt.$$

To calculate the probability that no splitting event happens in the interval $\left[t_{0},t_{0}+u\right]$ we integrate and get

(6) $$f_{d}(u|\mu ,\sigma ,t_{0})=e^{-\int_{t_{0}}^{t_{0}+u}\lambda \prime_{d}(t_{0}+t)dt}\lambda \prime_{d}(t_{0}+u)$$

(7) $$=e^{-\lambda_{d}(t_{0},t_{0}+u)}\lambda \prime_{d}(t_{0}+u)$$

where

(8) $$\lambda_{d}(t_{0},t_{0}+u)=\text{log}\;\left(\frac{\text{erf}\left(\frac{\mu -t_{0}}{\sqrt{2}\sigma }\right)-\text{erf}\left(\frac{\mu -b_{1}}{\sqrt{2}\sigma }\right)}{\text{erf}\left(\frac{\mu -(t_{0}+u)}{\sqrt{2}\sigma }\right)-\text{erf}\left(\frac{\mu -b_{1}}{\sqrt{2}\sigma }\right)}\right).$$

Combining these individual waiting times for coalescence, immigration, and splitting leads to the overall probability density for the waiting time *u* to the next event in the interval $\left[t_{0},t_{0}+u\right]$

(9) $$f(u|\Theta ,M,\mu ,\sigma ,t_{0})=e^{-u\lambda_{c+m}-\lambda_{d}(t_{0},t_{0}+u)}\left(\lambda_{c+m}+\lambda \prime_{d}(t_{0}+u)\right).$$

### Genealogy-probability calculations

The posterior density distribution p(ρ|D) for all parameters *ρ* given the data *D*, such as mutation-scaled population size Θ, mutation-scaled immigration rates *M*, divergence time mean *μ*, and standard deviation *σ*, is

(10) $$p(\rho |D)=\frac{p(\rho )\int_{G}f(G|\rho )p(D|G)dG}{p(D)}.$$

The genealogy *G* is an ultrametric tree with branch lengths augmented with migration and divergence events. We approximate the posterior distributions with histograms for each parameter *ρ* collected through the Markov chain Monte Carlo run. The Metropolis-Hastings acceptance/rejection steps will need calculations of the likelihood of the genealogy p(D|G) and the parameters p(G|ρ) (Beerli and Felsenstein 1999; Beerli 2006). The likelihood of the genealogy is calculated using the familiar likelihood pruning algorithm used in phylogenetics (Felsenstein 1981). We discuss the evaluation of p(G|ρ) in the next section.

### Probability of events and calculation of the probability density of a genealogy given all parameters

The coalescence process reduces the number of lineages when looking backward in time; coalescent, migration, and divergence events are independent from the events before them. Thus, we can calculate the probability density of a genealogy *G* given all parameters f(G|ρ) as the product over all time intervals, with ρ=θ,M,μ,σ, we get

(11) $$f(G|\rho )=\prod_{i}^{I}p(t_{i}-t_{i-1}|G,t_{i-1},\rho ).$$

The calculation of $p(t_{i}-t_{i-1}|G,t_{i-1},\rho )$ is more involved. For each time interval, we calculate the exponential waiting time for any event, calculate the probability that the particular event type recorded on the genealogy is drawn, and also need to adjust for how many possible events of the same type can be drawn. If we have a time interval that ends with a coalescent event, then, in the most general case, we calculate

(12) $$\begin{array}{c} p(t_{1}-t_{0},{\text{event}}_{c}|G,t_{i-1},\rho )=\lambda (t_{0},t_{1})e^{-\int_{t_{0}}^{t1}\lambda (t_{0},t)dt}\xi_{c}\frac{1}{\left(\begin{array}{c} k\\ 2 \end{array}\right)}\\ \xi_{c}=p(t_{c}<t_{m}\wedge t_{c}<t_{d}) \end{array}$$

where $\lambda (t_{0},t_{1})$ is the sum of all rates for all parameters, for example this includes Equations (1), (2), and (4). There are similar formulae for cases when the interval ends with a divergence event or ends with an immigration event. In a model with only coalescence and migration events, this simplifies greatly because $\xi_{c}=p(t_{c}<t_{m}\wedge t_{c}<t_{d})$ and reduces to $p(t_{c}<t_{m})$ because divergence events are not present. Details of this evaluation are described in the supplement (http://github.com/pbeerli/divergencesupplement). Including a hazard function that changes with time *t* for the divergence parameters leads to a more complicated situation. The probability that a divergence event comes before a coalescent or a migration event is

(13) $$\begin{array}{c} \xi_{d}=p(t_{d}<t_{c}\wedge t_{d}<t_{m})\\ =\int_{0}^{\infty }p(t_{c}>t_{0}+u)\;p(t_{m}>t_{0}+u)\;\lambda \prime_{d}(t_{0}+u)f_{d}(u|\mu ,\sigma ,t_{0})du. \end{array}$$

The $t_{d}=t_{0}+u$ is the time of a divergence event, *t_c_* is the time of a coalescent event, and *t_m_* is the time of a migration event. The $\lambda \prime_{d}(t_{0}+u)$ and $f_{d}(u|\mu ,\sigma ,t_{0})$ are defined in Equations (4) and (6). Since $p(t_{c}>t_{0}+u),\;p(t_{m}>t_{0}+u)$ become $e^{-u\lambda_{c}}$ and $e^{-u\lambda_{m}}$, respectively, we can write

(14) $$\xi_{d}=\int_{0}^{\infty }e^{-u\lambda_{c+m}}\lambda \prime_{d}(t_{0}+u)\;e^{\lambda_{d}(t_{0},t_{0}+u)}du.$$

Unfortunately, the integral in Equation (14) and its equivalents, *ξ_c_* and *ξ_m_*, cannot be solved analytically. The problem stems from the time dependence of the divergence rate $\lambda \prime_{d}(t_{0}+u)$. Looking for a faster way to compute these quantities, we use an approximation. Instead of solving the integral in Equation (6) numerically, we approximate using the midpoint rule. We replaced the midpoint $t_{0}+u/2$ with a fixed value $t_{0}+\epsilon$ where epsilon≤u:

(15) $$\begin{array}{c} f_{d}(u|\mu ,\sigma ,t_{0})=e^{-\int_{t_{0}}^{t_{0}+u}\lambda \prime_{d}(t)dt}\lambda \prime_{d}(t_{0}+u)\\ \approx e^{-u\lambda \prime_{d}(t_{0}+\epsilon )}\lambda \prime_{d}(t_{0}+\epsilon ). \end{array}$$

This approximation leads to a simpler formulation of Equation (14) which now becomes:

(16) $$\xi_{d}\approx \int_{0}^{\infty }e^{-u\lambda_{c+m}}\lambda \prime_{d}(t_{0}+\epsilon )\;e^{-u\lambda \prime_{d}(t_{0}+\epsilon )}du.$$

All *λ_i_* are constant with respect to *du* and, therefore, the integral can be solved using the substitution rule, and we get

(17) $$\xi_{d}\approx \frac{\lambda \prime_{d}(t_{0}+\epsilon )}{\lambda_{c+m}+\lambda \prime_{d}(t_{0}+\epsilon )}.$$

**Fig. 2.**
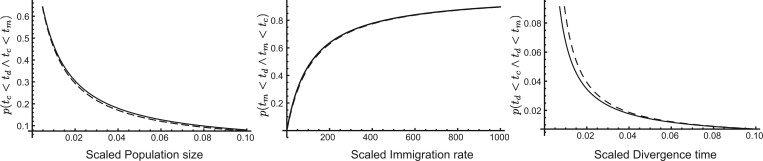
Numerical comparison of the full integral solution 14 (dashed curves) and the midpoint approximation 17 (black lines) of probabilities of occurrence for (from left) mutation-scaled population size *θ* (with fixed parameters $M=100,\mu =0.005,\sigma =\mu ,t_{0}=0$), mutation-scaled immigration rate *M* (with $\theta =0.01,\mu =0.005,\sigma =\mu ,t_{0}=0$), and mutation-scaled divergence time *μ* (with $\theta =0.01,M=100,\sigma =\mu ,t_{0}=0$).

A comparison for different values of *θ*, *M*, and divergence times *μ* shows that the integral 14 and the ratio 17 lead to very similar values (Fig. 2).

This result simplifies Equation (12) for the coalescent with divergence and migration. The general equation for all events becomes

(18) $$p(u|G,t_{0},\rho )=e^{-u(\frac{k(k-1)}{\Theta }+kM+\lambda_{d}(t_{0},t_{0}+\epsilon ))}\{\begin{array}{cc} \frac{\lambda \prime_{d}(t_{0}+\epsilon )}{k} & \left(\text{divergence}\right)\\ \frac{2}{\Theta } & \left(\text{coalescent}\right)\\ M & \left(\text{migration}\right) \end{array}.$$

These formulas are used in Equation (11) to calculate the probability of a genealogy given all the parameters f(G|ρ). The topology and all the times of all the events are fixed in this genealogy, so we can use the time interval *u* between events to replace *ϵ*.

The exposition in the section used only one rate for each of the event types; in reality, there may be many rates for each type; for example a model with 2 contemporary populations, 1 ancestral population, and gene flow among the contemporary populations will lead to 3 coalescent rates, 2 immigration rates, and 1 rate for the divergence time and its standard deviation.

### Implementation

The approach was implemented into the program *Migrate* (Beerli 2006). New parameter values were drawn from prior distributions, for example from an exponential distribution with fixed mean or a uniform distribution with a lower and upper bound. The genealogy-change proposal was described by Beerli and Felsenstein (1999), the procedure remains the same except that for the proposal of a new event and its time. In earlier versions of *Migrate*, the time was drawn by solving formula (3) for the time interval *u* using a random number on the interval (0,1] as the probability, and then the probability of a particular event at that time $t_{0}+u$ was calculated. The hazard function for the splitting rate added considerable complexity. Instead of proposing a time for any possible event and then choosing among events, we propose a time for each possible event independently and pick the event that comes first. For example, the proposed interval *u* of the splitting time using formula (8) is

(19) $$u=\mu -t_{0}-\sqrt{2}\;\sigma \;{\text{erf}}^{-1}\left(\text{erf}(\frac{b_{1}-\mu }{\sqrt{2}\;\sigma })(r-1)+\text{erf}(\frac{\mu -t_{0}}{\sqrt{2}\;\sigma })\;r\right).$$

Thus, for every change of the genealogy we need to propose times for coalescence, migration, and divergence events. Among these times, we pick the event with the shortest time. This approach allows us to draw the events at the correct frequency without calculating the complex ratio described in the earlier section; both, the earlier and this new calculations take about the same amount of time.

In contrast to other programs, *Migrate* does not need a specific guide tree to specify the order of the splitting events. It uses an extension of the adjacency matrix introduced into *Migrate* in 2001 (Beerli and Felsenstein 2001). This matrix defines the connections among the populations by migration events and or divergence events. It can specify particular divergence models without the need to define the order of the splitting times; for example for a model in which 2 island populations were colonized independently from a mainland population, *Migrate* does not force the user to specify an order of the time of the colonization events. We caution that our approach is not equivalent to exploring all possible population trees. Comparisons of different population trees are possible by treating each population tree as a new hypothesis and run each of these hypotheses independently, followed by Bayesian model comparison (Beerli and Palczewski 2010; Palczewski and Beerli 2014). Tutorials, source code and executables can be found on the *Migrate* website (http://popgen.sc.fsu.edu).

### Simulation

**Fig. 3.**
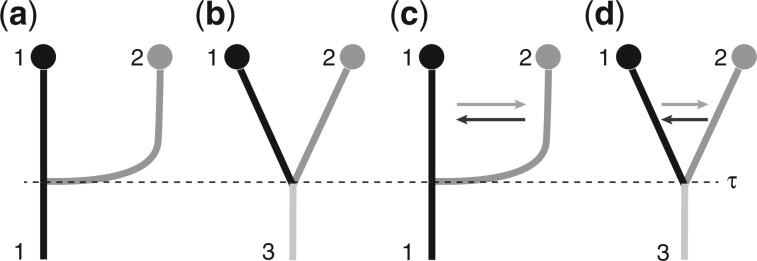
Simulation and analysis scenarios: a) population 2 splits off from population 1; b) the ancestral population 3 splits into 2 contemporary populations; c and d) immigration. Models (a) and (c) were used to simulate data. Models (a) and (c) use population 1 as being ancestral and present-day. Models (b) and (d) have an additional parameter for ancestral population size.

Simulations were performed over a wide range of (true) divergence times *τ* from $1/512\times N_{e}$ to $8.0\times N_{e}$ generations between 2 populations with a combined size of Θ=0.02; each population had 20 samples; each simulated locus had 1,000 bp. We performed 3 sets of simulations. The first simulation set explored the accuracy of a simple divergence model (Fig. 3a). For each divergence time, 4 replicate datasets were simulated using the program *ms* (Hudson 2002) to generate the genealogies. Our own program *migdata* (available on github.com/pbeerli/popsimulate.git) used these genealogies to generate sequence data. To explore effects of the number of loci, we generated 2- and 10-locus datasets for all divergence times. For a subset of divergence times, we also generated 1,000-locus datasets. The second simulation set explored the interaction of immigration and divergence. We used a scenario with 2 populations exchanging 1 migrant every 16 generation, 4Nm=0.25, and 1 migrants every 4 generations, 4Nm=1.0, respectively (Fig. 3c). The immigration numbers guarantee the long-term maintenance of population structure. The third simulation set explored the effect of the estimation of the standard deviation of the divergence time. We ran simulations using our own simulator *speciessim.py* (available on github.com/pbeerli/speciessimulate) using the same setting of the divergence times as before, but changed the standard deviation of the divergence time to values of $\sigma =\tau /10^{4},\;\sigma =\tau /2$, and σ=τ for datasets of 10 loci and compared these with the simulations of *ms* which simulates divergence times only with σ=0.

All datasets were analyzed with *Migrate* using the Felsenstein 1984 finite mutation model for all runs. We picked an exponential prior for the mean of the divergence time distribution with an upper bound that was 3× larger than the oldest divergence time simulated; the same prior was used for all different divergence times; in preruns, we established that the prior choice does not change the general results. Our choice of prior and its width was done so that we could run all simulations with as little changes of options as possible. For run with real data we suggest that the range of the priors are evaluated with test runs, the divergence time *μ* in *Migrate* is scaled by mutations; it is on the same scale as the mutation-scaled population size Θ. The choice of the prior for the standard deviation is more consequential when immigration is coestimated, large prior ranges interact with immigration-rate estimation; consequently we picked a small prior range for the standard deviation for the second set of simulations and also for the comparison with other programs. Simulation were run on a computer cluster at the Florida State University Research Computing Center with various number of computer cores, the 10-locus simulations were run on 21 computer cores. After experimentation with run-length we established that runs longer than 15 min are sufficient for our simulation data sets. The 1,000-locus datasets were run on 501 cores and took about 3 h 20 min.

### Comparison with other program estimating divergence times among populations

We compared our approach with *IMa2p* (Sethuraman and Hey 2016), *BPP* (Yang and Rannala 2010), and *Momi2* (Kamm et al. 2020) using simulated data from population models shown in Fig. 3, a and c with immigration rates of 4Nm=0.0,4Nm=0.25,4Nm=1.0 and divergence times of $[0.125,0.5,1.0,2.0,3.0,4.0,5.0,6.0,7.0,8.0]\times 4N_{e}$ generations. The run conditions for all programs are available in the electronic supplement and the data converter from the *Migrate* format to the other programs is available from https://github.com/pbeerli/dataconverters.

### An example using samples of human populations

We showcase the potential of our method reanalyzing a dataset of modern and archaic human populations. A larger dataset that includes our samples was analyzed by Kamm et al. (2020). Preliminary analyses with *Migrate* suggested that fitting a very complex model with only a few individuals may be prone to overfitting and difficult to interpret. Therefore, we decided to prune the problem to an analysis that is simpler and also easier to judge whether the results are appropriate. We used the VCF data of chromosome 21 provided by Jack Kamm. We analyze all SNPs (*n *=* *131,249) as linked SPS on loci of length of 100,000 bp. This lead to a data set of 336 loci (dataset is available at http://github.com/pbeerli/divergencesupplement). The resulting dataset consisted of 5 populations with a total of 9 individuals: 3 present-day populations (3 Mbuti, 2 Sardinians, 2 Han), and 2 ancient DNA samples: the Altai Neanderthal and modern human from Ust’Ishim, Siberia.

## Results

### Simulation

#### Splitting time estimation

**Fig. 4.**
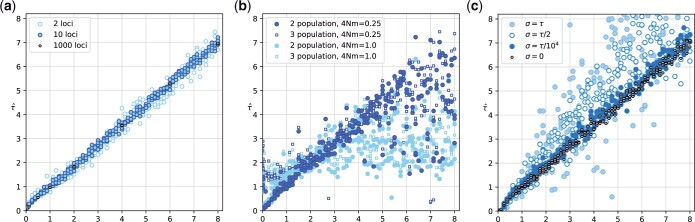
Comparison of estimated divergence time $\hat{\tau }$ with the true divergence time *τ_T_*. a) Results of 2-, 10-, and 1,000-locus data. The data were simulated and analyzed using the model shown in Fig. 3a. Units of $\hat{\tau }$ and *τ_T_* are in $N_{e}\times$ generations. b) The data were simulated using the model shown in Fig. 3c, and analyzed using models Fig. 3, c and d. The number of immigrants per generation was 4Nm=0.25 and 4Nm=1.0, respectively. Units of $\hat{\tau }$ and *τ_T_* are in $4N_{e}\times$ generations. c) The 10-locus data were simulated without immigration but with 4 different standard deviations for the splitting time ($\sigma =\tau ,\sigma =\tau /2,\sigma =\tau /10^{4}$, and σ=0).

The inference code was evaluated using simulations over a wide range of divergence times and 2 different migration rates after the population split. Figure 4a compares the estimated population split time $\hat{\tau }$ with the population split time *τ* used to simulate the data without migration. The estimates track the simulated split times well, although the estimates of large divergence times are underestimated. As expected, the estimates from 2-locus data sets show more spread than those from 10- or 1,000-locus datasets. A comparison of the 95% credibility intervals of runs with 2, 10, and 1,000 loci shows this trend: standardizing the credibility interval with the observed mode ($\frac{p_{97.5\%}-p_{2.5\%}}{p_{\text{mode}}}$) leads to averages of 2.48 for 2 loci (N=280,std=1.21), 1.07 for 10 loci (N=280,std=1.29), and 0.47 for 1,000 loci (N=23,std=0.59).

#### Splitting time estimation under the isolation with migration model

The simulation results with migration deliver a more complicated message. Simulations with low recurrent immigration rates (4Nm=0.25) during the time interval from today to the population split track the true population split often quite well but has a considerable fraction of runs that underestimate the divergence time (Fig. 4b). With higher immigration rate (4Nm=1.0), $\hat{\tau }$ underestimates the true divergence time of datasets that were created using a high divergence time and overestimates the divergence time for very recent divergences.

#### Effect of simulated uncertainty of splitting time

Our approach allows the estimation of the splitting time and the standard deviation of the splitting time. All current simulation methods, except our own *speciessim.py* simulator, assume a defined time when the ancestral population splits into offspring populations. Our simulation and estimation model allow uncertainty about this time, a small standard deviation, such as σ=1/10,000, will result in simulated data sets that mimic the standard simulation method in *ms*. Large standard deviation leads to datasets with skewed distributions of divergence times because the divergence time cannot be negative (looking backward in time), and such times had to be redrawn to generate the simulated genealogies. About 15.8% of all random draws from a Normal distribution will be smaller than τ−σ. This resulted in datasets that come from older divergence times on average and will result in higher divergence time estimates than the divergence time *τ* used to generate the datasets (Fig. 4c).

#### Exploration of the splitting time bias in the IM model

**Fig. 5.**
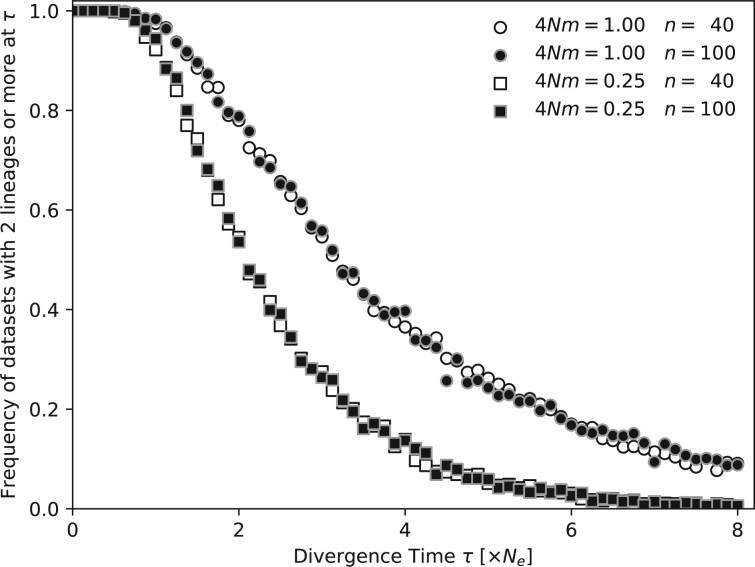
Percentages of simulated datasets with 2 or more lineages in the sample at divergence time *τ*. For each divergence time *τ* 1,000 datasets were simulated.

If the immigration rate is high, population divergences that happened far in the past are problematic to estimate because in comparisons (Fig. 4b) we detect a bias toward more recent split time estimates than those simulated. To investigate this bias, we have simulated genealogies with sample sizes of 40 and 100 with the same parameters used to create the data reported in Fig. 4b and recorded the number of lineages present at the time of population split (70 time points between split times of *τ* from $1/512\times 4N_{e}$ to $8.0\times 4N_{e}$ generations). Figure 5 reports the percentage of datasets that have 2 or more lineages available at these 70 time points (*N* = 1,000 for each time point). The graphs for 4Nm=0.25 (low) and 4Nm=1.0 (high) differ starkly in the percentages with high divergence times. With high immigration rates (4Nm=1.0) the chance of having the sample coalesced to a single lineage increases considerably. For example, fewer than 20% of the datasets have information about a divergence time of $4N_{e}$ generations. Once a sample coalesces into a single lineage all information about the historical processes is lost and any inferred result will only come from the prior, thus is independent of the data. Increasing the number of individuals from 40 to 100 for each dataset does not improve the number of available lineages at the divergence time. With low immigration rates, the time of the most recent common ancestor is beyond the divergence time; thus, the remaining lineages may have information about the splitting time.

#### Comparison with other programs

**Fig. 6.**
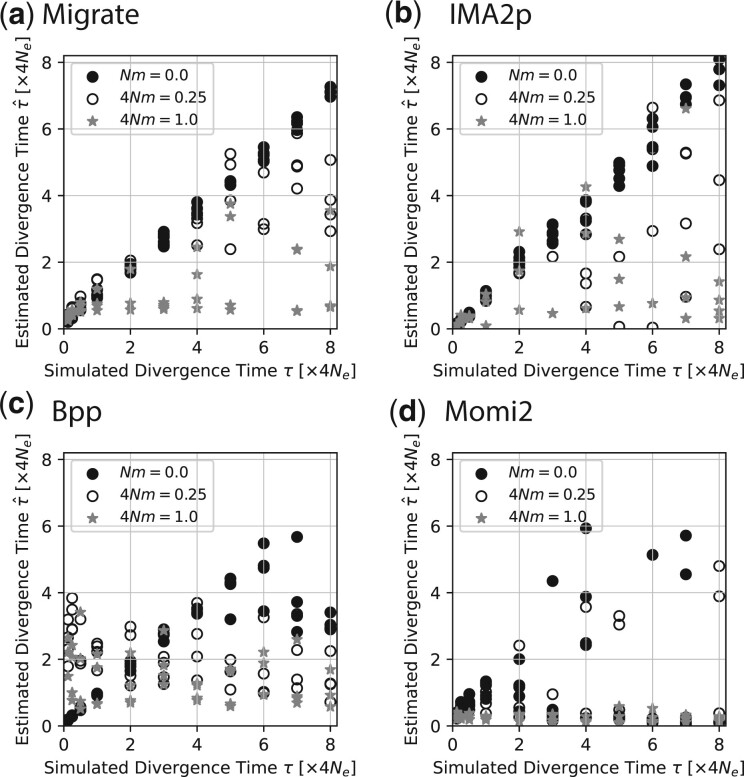
Comparison of estimated divergence time $\hat{\tau }$ and the true *τ_T_* for a) *Migrate*, b) *IMa2p*, c) *BPP*, and d) *Momi2*. The data were simulated using the model shown in Fig. 3c and analyzed using models Fig. 3, c and d. The number of immigrants per generation was 4Nm=0.0, 4Nm=0.25, and 4Nm=1.0, respectively.

Three sets of simulated data for immigration rates of 4Nm=[0.0,0.25,1.0] were used to compare the results of 4 different programs: *Migrate*, *IMa2p*, *BPP*, and *Momi2*. Figure 6 shows the results for these comparisons. Divergence times can be well estimated by all programs when recurrent gene flow is zero and the true divergence time is smaller than $2N_{e}$ generations. All programs show a bias when the true divergence times become large compared to the population size of the sampled populations, *Migrate* and *IMa2p* in particular show a smaller bias than the others. Results become more unpredictable when gene flow is larger than zero. *Migrate* and *IMa2p* become more variable in their estimates, but for many datasets with large true divergence times the estimates reveal also large divergence times. *BPP* and *Momi2* fail to recover divergence time with recurrent gene flow; both program are not designed to estimate divergence time under recurrent gene flow but only allow for pulses of gene flow. With immigration rates of 4Nm=1.0, all programs fail to estimate accurate divergence times. *Migrate* and *IMa2p* deliver very similar results. Both are overestimating divergence times when the true divergence is small and underestimating when the true divergence times are large.

**Fig. 7.**
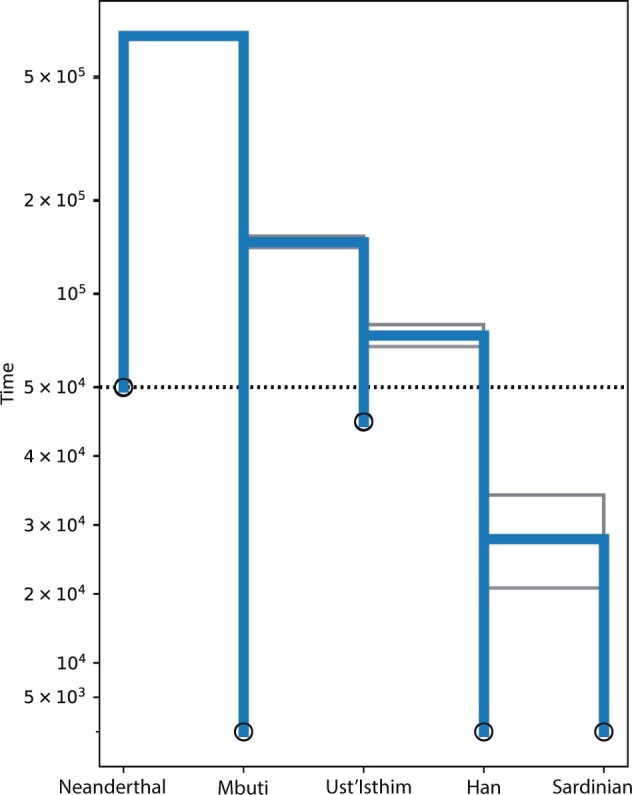
Population splitting among 5 populations. Three populations were sampled at present time whereas 2 populations are archaic. The gray lines mark 50% credibility interval for each divergence time. The scale of the *Y*-axis is linear below the dashed line and logarithmic above.

### Application to a human dataset

The 5 population dataset was originally run on a large cluster computer using 336 compute nodes, 1 for each locus. After recognizing that a 100x shorter run delivers the same results we ran our test cases on a Macbook Pro 2020 using 8 cpu-cores with runtimes under 30 min. The pattern of the divergence times are not surprising (Fig. 7), perhaps except that in our experiments the population model that splits the archaic Ust’Ishim from the African Mbuti an then the Han Chinese population from the Ust’Ishim had a much higher marginal likelihood (Beerli and Palczewski 2010) than models that suggested that both, the Ust’Ishim and the Han, independently split from the Mbuti. A comparison of our result with *Momi2* revealed that the divergence time estimates depend on the assumption of the overall population size. Our approach and also *Momi2* estimated about 100,000 individuals for each populations which seems high but since *Migrate* is not estimating the mutation rate, this may be an artifact of the application of an independent mutation of $1.25\times 10^{-8}$ per generation. Using a total population size of 250,000 *Momi2* delivers similar divergence estimates (for a table with the parameter estimates see http://github.com/pbeerli/divergencesupplement).

## Discussion

We have developed a model to incorporate population splitting and population admixture. Our algorithm differs from other algorithms because we treat the splitting times as random variables with truncated Normal distributions. This method allows a wide range of analyses, such as having populations split from an ancestral population or having population split from a population that is the same today and in the past.

The joint estimation of divergence time and population sizes without immigration from large genetic datasets seems feasible with little error. Our simulations assumed informative loci and no complications with the finite mutation model. However, *Migrate* can handle site rate variation and more complex mutation models than the F84 model used in the simulations. We assume that *Migrate* has similar vulnerabilities as IM when tested with deviations of the model (cf. Strasburg and Rieseberg 2010).

*Migrate* runs each locus as an independent unit and thus can easily run large datasets, such as the 1,000 loci datasets used in the simulations, in reasonable time on a cluster computer: the 1,000-locus datasets for Fig. 4a were run on 501 computer cores and finished in about 3–4 h. The comparisons of the data with 2, 10, and 1,000 loci show that with informative loci, we may not need to have many loci to extract the most likely parameter value, although the standard deviations of the 1,000-locus runs are smaller than the 2 or 10 locus datasets.

It seems straightforward to use an immigration with divergence model (IM; Nielsen and Slatkin 2000), but little exploration about the power of the inference has been conducted. Strasburg and Rieseberg (2010) highlighted that assumption misspecification can lead the program IM (Hey 2010) to deliver biased answers. Recently, Quinzin et al. (2015) evaluated the program IM and observed that divergence time estimates are more accurate if migration is low and if the populations are large compared to the divergence time. We find similar patterns with *Migrate* and *IMa2p*. In addition, our coalescent simulations with migration show a deeper problem with such inferences, even when assumptions are met. Looking backward in time, once all samples have coalesced, no information is left to estimate parameters. In a model with immigration and population splitting there has to be a balance so that we can see the effects of one or the other: if the migration rates are very small, then all sample lineages, looking backward in time, will have joined the ancestral population before having experienced a migration event. In contrast, with high immigration rates, it becomes very likely that all lineages have coalesced into 1 lineage before the expected splitting time. Figure 5 shows that many sample data never experience a population split. It will certainly be difficult to estimate an event that did not leave a trace in the sample. Hence, the estimated divergence times will not reflect the true splitting time and will be too close to the sampling date. However, with small immigration rates it is possible to recover splitting times that are further in the past (Fig. 4b). The same simulations also show that it is unproblematic to estimate splitting times that are old when there is no immigration. The results for *Migrate* and *IMa2p* that use recurrent gene flow in their models contrast considerably with the results of *BPP* and *Momi2* that model geneflow using pulses of migrants. It seems important to highlight this model difference when describing results of these programs. Our results for *Migrate*, *IMa2p*, *BPP*, and *Momi2* suggest that one should use caution when using models with immigration and population splitting times. This dependency is independent of the estimation method and certainly will include other than the tested methods, too. We believe that this dependency has and will lead to incorrect reports of divergence times: divergence times are reported to be more recent than they really are.

The ancient human dataset D1 is based on transversions only. Our method can analyze complete sequences taking into account average base frequencies of the data and finite mutation models; here, we simply used the F84 model, but more sophisticated models such as Tamura-Nei are possible; currently, we do not know of a good comparison of site-frequency-based methods and finite-mutation models in a genomic context. We acknowledge that our method becomes very time consuming with large number of loci and also large numbers of samples. The use of large computer clusters allowing to run independent loci in parallel helps to analyze such problems.

We have presented an alternative to current estimations of divergence time among populations. Our method not only allows considering the splitting times but even allows to date admixture of a population from 2 or more ancestral populations. The simulations suggest that fairly variable data are needed. Estimation of splitting times alone is robust over a wide range of simulated splitting times, whereas models that allow migration and splitting times (IM model) simultaneously suffer considerable difficulties estimating splitting times that are far in the past when population sizes are small and immigration rates are high. These difficulties are caused by the sparsity of lineages far in the past, a situation that is well known (Heled and Drummond 2008). Improving these estimates will depend on the number of loci, the number of individuals, and data with different sampling dates.

## Data availability

The code to recreate the simulated datasets and the data for the human example data are available in the public repository on http://github.com/pbeerli/divergencesupplement. Original simulation datasets can be supplied on request. An elaboration on some of the equations is available from http://github.com/pbeerli/divergencesupplement. The software *Migrate* is available at the *Migrate* website http://popgen.sc.fsu.edu, and simulation software is available at http://github.com/pbeerli/popsimulate and http://github.com/pbeerli/speciessimulate.
